# VE/VCO_2_ slope and its prognostic value in patients with chronic heart failure

**DOI:** 10.3892/etm.2015.2267

**Published:** 2015-02-05

**Authors:** YUQIN SHEN, XIAOYU ZHANG, WENLIN MA, HAOMING SONG, ZHU GONG, QIANG WANG, LIN CHE, WENJUN XU, JINFA JIANG, JIAHONG XU, WENWEN YAN, LIN ZHOU, YI NI, GUANGHE LI, QIPING ZHANG, LEMIN WANG

**Affiliations:** 1 Department of Cardiology, Tongji Hospital Affiliated to Tongji University, Shanghai 200065, P.R. China; 2 Department of Rheumatology, Tongji Hospital Affiliated to Tongji University, Shanghai 200065, P.R. China

**Keywords:** heart failure, cardiopulmonary exercise test, ventilatory expired gas

## Abstract

The minute ventilation/carbon dioxide production (VE/VCO_2_) slope has been widely demonstrated to have strong prognostic value in patients with chronic heart failure (CHF), and the risk of mortality is believed to increase when the VE/VCO_2_ slope is >32.8; however, there is little evidence concerning the prognostic value of the VE/VCO_2_ slope in Chinese patients. In the present study, the prognostic value of the VE/VCO_2_ slope was investigated in patients with CHF. A total of 258 subjects underwent symptom-limited cardiopulmonary exercise testing (CPET) and were divided into CHF (113 males and 16 females; LVEF <0.49) and control (106 males and 23 females) groups. The cardiac-related events over a median 33.7-month follow-up period subsequent to the CPET were evaluated using receiver operating characteristic curve analysis. The VE/VCO_2_ slope was significantly different between the CHF and control groups (P<0.001). The area under the curve (AUC) for the VE/VCO_2_ slope in predicting cardiac-related mortalities in the patients with CHF was 0.670 (P<0.05), and the sensitivity and specificity of the VE/VCO_2_ slope were 0.667 and 0.620, respectively. The optimal threshold of the VE/VCO_2_ slope for predicting cardiac-related mortalities in patients with CHF was ≥39.3. The AUC for the VE/VCO_2_ slope in predicting cardiac-related hospitalizations in patients with CHF was 0.682 (P<0.05), and the sensitivity and specificity of the VE/VCO_2_ slope were 0.631 and 0.778, respectively. The optimal threshold of the VE/VCO_2_ slope for predicting cardiac-related hospitalizations in patients with CHF was ≥32.9. In conclusion, ventilatory efficiency decreases in patients with CHF. The VE/VCO_2_ slope is a strong predictor of cardiac-related mortalities in the patients with CHF analyzed.

## Introduction

The minute ventilation/carbon dioxide production (VE/VCO_2_) slope reflects the increase in ventilation in response to CO_2_ production, and thus shows increased ventilatory drive (1). Changes in the VE/VCO_2_ slope may be induced by increases in the number of chemoreceptors, the peripheral ergoreceptor response, the ventilatory dead-space and also by the muscle mass engaged in exercise (2–5). Arena *et al* (6) reported that based on the VE/VCO_2_ slope, there is a 4-level ventilatory classification (VC) system (VC-I, ≤29.9 implies negligible risk of a major cardiac event; VC-II, 30.0–35.9, low risk of major cardiac event; VC-III, 36.0–44.9, moderate risk of major cardiac event; and VC-IV, ≥45.0, high risk of major cardiac event) which is currently used in clinical practice. Other indices, including peak oxygen consumption rate (VO_2_), anaerobic threshhold VO_2_ and the oxygen uptake efficiency slope, can be used to indicate the prognosis for cardiac-related events. Studies have shown that the VE/VCO_2_ slope exhibits a high prognostic value for cardiac-related events in patients with chronic heart failure (CHF) (6–11), and the risk of mortality is believed to increase when the VE/VCO_2_ slope is >32.8 (12). The VE/VCO_2_ slope also provides useful information for the management of CHF. There is, however, no evidence concerning the VE/VCO_2_ slope and its prognostic value for cardiac-related events in Chinese patients, and there are no studies suggesting different VE/VCO_2_ slope prognostic values for cardiac-related events in Chinese patients with CHF versus non-Chinese subjects.

In China, the estimated prevalence of heart failure is 0.9%, and there are ~4 million patients with CHF. Furthermore, the number of patients is increasing annually (13). High morbidity and mortality rates, recurrent hospitalization and a heavy medical burden are common concerns in patients with CHF around the world. The aim of the present study was to investigate ventilatory efficiency by analyzing the VE/VCO_2_ slope in Chinese patients with CHF and to assess the prognostic value of the VE/VCO_2_ slope in this population.

## Subjects and methods

### Subjects

Patients with CHF were recruited from the Department of Cardiology of the Affiliated Tongji Hospital of Tongji University (Shanghai, China) between August 2007 and May 2013. The inclusion criteria consisted of a diagnosis of heart failure (14) and evidence of left ventricular systolic dysfunction on two-dimensional echocardiography obtained within one month of cardiopulmonary exercise testing (CPET) [patients in the registry with a left ventricular ejection fraction (LVEF) of ≥49% were excluded from the analysis]. Patients with a diagnosis of significant pulmonary disease (maintained on home oxygen therapy for lung disease and/or inhaled corticosteroids) were excluded from the study. A total of 129 patients (113 males and 16 females) with a mean age of 59.1±11.4 years were enrolled into the CHF group. The mean body mass index (BMI) of the patients was 24.7±3.7 kg/m^2^ and the LVEF was 0.38±0.09%. The cardiac function of the patients was classified according to the New York Heart Function Assessment (NYHA) as grades I–III (NYHA I, n=5; NYHA II, n=68; NYHA III, n=56) (15). Among the 129 patients, 74 were diagnosed with coronary artery disease with indication for cardiac catheterization, and 55 with dilated cardiomyopathy. During the study, CHF therapy was allowed to continue with digitalis (43.0%), β-blocker (89.0%), angiotensin-converting enzyme inhibitor (ACEI) and angiotensin II receptor blocker (91.0%) and diuretics (51.0%). One day before the CPET, treatment with digitalis, β-blocker, ACEI, angiotensin receptor blocker or diuretics was discontinued, but these treatments were re-initiated following the CPET.

In addition to the CHF group, healthy volunteers were screened for inclusion in a control group. Volunteers were excluded if they had been diagnosed with a chronic illness or were receiving chronic medication; if they had any current health complaints, an abnormal physical examination (including a blood pressure of ≥140/90 mmHg) or abnormal results on the screening tests [electrocardiogram (ECG), rest and exercise echocardiogram and spirometry]; or if they were participating in regular exercise. A total of 129 healthy age-matched volunteers (106 males and 23 females) met these criteria and were enrolled. The gender and body mass index (BMI) of the volunteers were also matched. Informed consent was obtained from all patients prior to recruitment in accordance with the protocol approved by the Ethics Committee of the Affiliated Tongji Hospital of Tongji University. The study was registered in the China Clinical Trial Registration Center (registration no. ChicTR-TRC-00000235).

### Echocardiography

Echocardiography was performed on each subject using the GE Vivid™ 7 EchoPAC™ system (GE Healthcare, Pittsburgh, PA, USA) with a high-definition 3.2-MHz transducer. All data were measured by the same qualified physician. Standard M-mode and two-dimensional echocardiography, as well as Doppler blood flow measurements, were performed in accordance with the American Society of Echocardiography guidelines (16). Interventricular septum thickness (IVST) and left ventricular posterior wall thickness (LVPWT) were obtained from the parasternal long axis view. The left ventricular end systolic diameter (LVESD) and left ventricular end diastolic diameter (LVEDD) were obtained from two-dimensional apical images. The LVEF was calculated from two-dimensional apical images according to the Simpson method (17), and the left ventricular mass (LVM) was calculated according to the formula proposed by Devereux *et al* (18): LVM (g) = 0.8 × [1.04 × (IVST + LVEDD + LVPWT)^3^ - LVEDD^3^] + 0.6. The LVM was subsequently adjusted for body surface area (BSA) to obtain the LVM index (LVMI): LVMI (g/m^2^) = LVM/BSA.

### CPET

The modified Ramp 10 protocol (19) was used to conduct the symptom-limited CPET with Variobike 500 exercise apparatus (GE Healthcare). Immediately prior to exercise testing the subjects were encouraged to produce a maximal effort. Analysis of the ventilatory expired gas was conducted using a metabolic cart (Innocur™ version 5.00, Breath-by-Breath; Innovision A/S, Odense, Denmark). Prior to each test, reference and calibration gases were used to calibrate the equipment according to the manufacturer’s instructions. Standard 12-lead ECGs were obtained at rest and during the exercise and recovery phases; blood pressure was measured using a standard cuff sphygmomanometer. Prior to exercising, the patients were asked to rest for 3 min. The exercise began with a 3-min warm-up at 0 W and 60–70 rpm, and thereafter a 10-W increment in load was administered every minute subsequent to exercising at 20 W for 2 min. Continuous ECG, manual blood pressure measurement and heart rate recording were performed every minute, and the Borg scale was used to rate the perceived exertion at each stage (20). Exercise was discontinued when the patients were physically exhausted, or developed severe dyspnea or dizziness. Several parameters were obtained through the metabolic systems during exercise testing, including oxygen consumption (VO_2_), VCO_2_ and VE. The anaerobic threshold was determined by the V slope (21). The VE/VCO_2_ slope was determined using linear regression analysis of VE and VCO_2_ obtained throughout the exercise period (22).

### End-points

The subjects were followed-up for a median of 33.7 months (maximum follow-up period, six years) for assessments of cardiac-related mortality and hospitalization following the CPET via medical chart review. Any mortality or hospitalization that occurred as a result of cardiac dysfunction, as per the hospital discharge diagnosis, was regarded as an event. The most common causes of mortality noted were cardiac arrest, myocardial infarction and end-stage heart failure. The most common causes of hospitalization were decompensated heart failure and coronary artery disease.

### Statistical analysis

All continuous data are presented as the mean ± standard deviation. Categorical variables are reported as percentages. The Student’s t-test and χ^2^ analysis were utilized to compare differences in the continuous and categorical variables, respectively. The NYHA grades were compared with a nonparametric rank-sum test. Receiver operating characteristic (ROC) and Cox regression analyses were employed to evaluate the predictive value of the VE/VCO_2_ slope for cardiac-related mortality and hospitalization. SPSS version 18.0 (SPSS Inc., Chicago, IL, USA) was used for the statistical analysis. A two-tailed P-value of <0.05 was considered to indicate a statistically significant difference.

## Results

### General patient data

Table I shows the characteristics of the patients with CHF and controls at baseline. No significant differences were found in the age, gender or BMI between the two groups; however, the LVMI was significantly higher and the LVEF was markedly lower in the patients with CHF when compared with those in the controls (P<0.01). The peak VO_2_ was significantly lower in patients with CHF compared with controls (P<0.01). The VE/VCO_2_ slope was significantly higher for the patients with CHF as compared with that for the controls (P<0.01). The patients with CHF (n=129) were followed-up for a median period of 33.7 months (maximum, six years), and 19 cardiac-related mortalities and 198 cardiac-related hospitalizations were recorded. The control group was followed up for a median period of 37.8 months (maximum, six years) and no cardiac-related mortalities or hospitalizations were recorded.

### Predictive value of the VE/VCO_2_ slope

**Figure 1:**
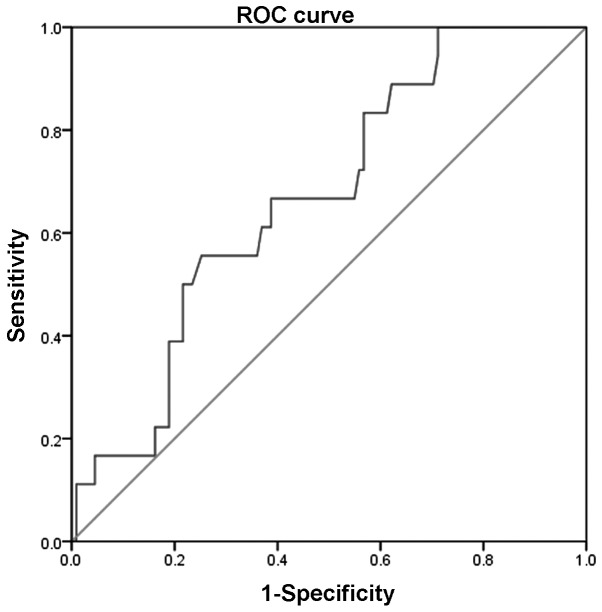
ROC curve depicting the sensitivity and specificity of the minute ventilation/carbon dioxide production slope in the prediction of cardiac-related mortalities in the chronic heart failure group. The area under the curve was 0.670 (P<0.05), and the sensitivity and specificity were 0.667 and 0.620, respectively. ROC, receiver operating characteristic.

**Figure 2:**
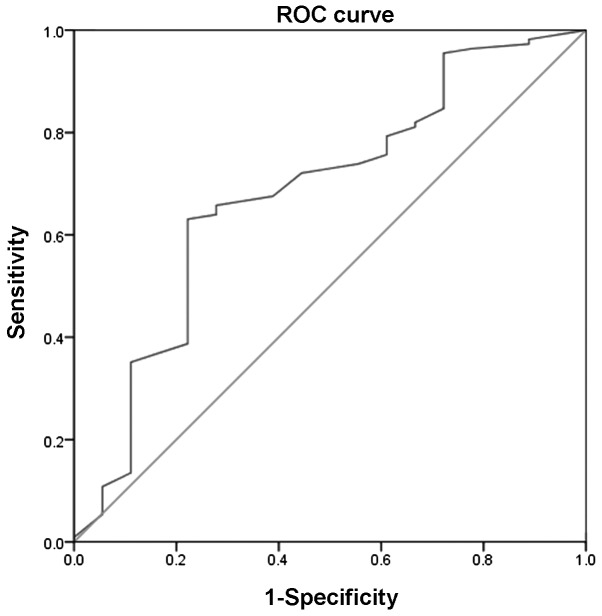
ROC curve depicting the sensitivity and specificity of the minute ventilation/carbon dioxide production slope in the prediction of cardiac-related hospitalizations in the chronic heart failure group. The area under the curve was 0.682 (P<0.05), and the sensitivity and specificity were 0.631 and 0.778, respectively. ROC, receiver operating characteristic.

**Figure 3:**
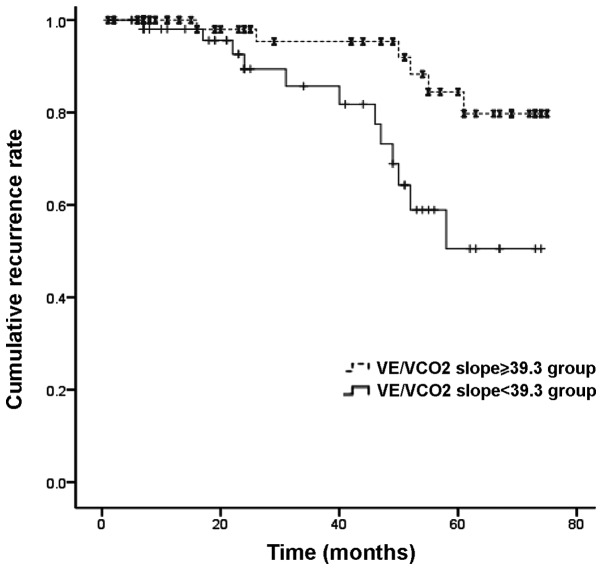
Kaplan-Meier survival curves between VE/VCO_2_ slopes of ≥39.3 and <39.3 in patients with chronic heart failure for the prediction of cardiac-related mortalities. Log-rank, 7.2; P<0.01. VE/VCO_2_, minute ventilation/carbon dioxide production.

**Figure 4:**
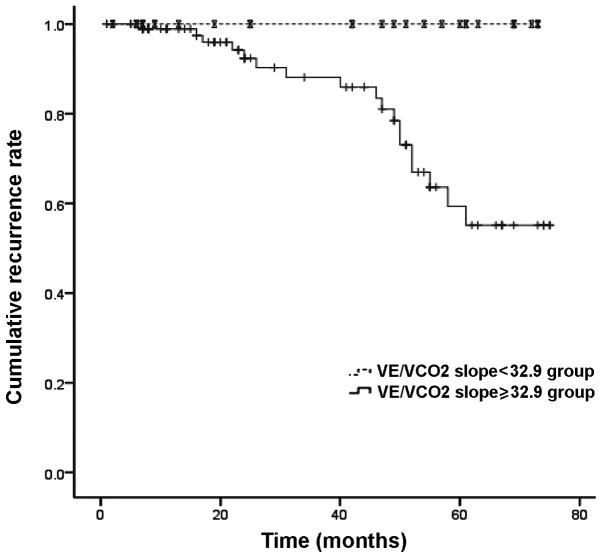
Kaplan-Meier survival curves between VE/VCO_2_ slopes of ≥32.9 and <32.9 in patients with chronic heart failure for the prediction of cardiac-related hospitalizations. Log-rank, 8.5; P<0.01. VE/VCO_2_, minute ventilation/carbon dioxide production.

ROC analysis was conducted to evaluate the predictive value of the VE/VCO_2_ slope for cardiac-related mortalities and hospitalizations in patients with CHF. The area under the curve (AUC) of the VE/VCO_2_ slope for predicting cardiac-related mortalities was 0.670 (P<0.05), and the sensitivity and specificity were 0.667 and 0.620, respectively. The optimal threshold of the VE/VCO_2_ slope for predicting cardiac-related mortalities was ≥39.3 in the patients with CHF. The AUC of the VE/VCO_2_ slope for predicting cardiac-related hospitalizations was 0.682 (P<0.05), and the sensitivity and specificity were 0.631 and 0.778, respectively. The optimal threshold of the VE/VCO_2_ slope for predicting cardiac-related hospitalizations was ≥32.9 in the patients with CHF (Table II, Figs. 1 and 2). The univariate Cox regression analysis showed that the optimal threshold of the VE/VCO_2_ slope was significantly correlated with the number of cardiac-related mortalities and hospitalizations (P<0.05) (Table III). The survival model showed the efficacy of the VE/VCO_2_ slope in predicting cardiac-related mortalities (Log-rank, 7.2; P=0.007; Fig. 3) and cardiac-related hospitalizations (Log-rank, 8.5; P=0.004; Fig. 4) in patients with CHF.

## Discussion

CHF is a common condition with high rates of morbidity and mortality and a high incidence of recurrent hospitalization around the world (23,24). With the increase in the prevalence of CHF in China (13), it is imperative to develop a simple and flexible measurement to provide more beneficial information for the management of CHF. CPET remains an important and objective means of assessing the functional capacity of patients with CHF. During CPET, peak VO_2_ and the VE/VCO_2_ slope during exercise are traditionally considered to be the major indices influencing the functional impairment and prognostic value. Exercise capacity, as reflected by peak VO_2_, has been consistently shown to be one of the most powerful prognostic markers in patients with CHF (11,25,26); however, the peak VO_2_ has important limitations and may not be accurately obtained, as it is susceptible to influence by the movement such that a plateau is not always achieved at the peak exercise. By contrast, the VE/VCO_2_ slope is easier to determine objectively than the maximal exercise capacity, and the VE/VCO_2_ slope is elevated in the majority of patients with CHF. Numerous studies have confirmed that the VE/VCO_2_ slope has equivalent or even superior prognostic value to the measurement of peak VO_2_ in patients with CHF (8,27–31).

The present results confirmed previous findings that the exercise ventilatory response was increased in the patients with CHF, as demonstrated by a higher VE/VCO_2_ slope. Patients who exhibit an increased ventilatory response to exercise also demonstrate a poorer exercise tolerance, as indicated by a reduction in the peak VO_2_ (32). Excluding the influence of matched factors, such as age, gender and BMI, the difference in the VE/VCO_2_ slope between the two groups was significant. By comparing the results of the two groups it was shown that the LVEF was lower, the LVMI and NYHA grade of cardiac function were higher and the peak VO_2_ was lower in patients with CHF as compared with the controls.

The present study demonstrated that the VE/VCO_2_ slope was a significant predictor of cardiac-related events in Chinese patients with CHF. The optimal threshold of the VE/VCO_2_ slope for predicting cardiac-related mortalities was ≥39.3 in the patients with CHF, which was consistent with the results in another study, in which the end-points were mortality, transplantation or left ventricular assist device implantation (optimal threshold of the VE/VCO_2_ slope, ≥45) (21). Furthermore, the optimal threshold of the VE/VCO_2_ slope for predicting cardiac-related hospitalizations was ≥32.9 in the patients with CHF. The present findings were also consistent with those in the study by Arena *et al* (33), which utilized the same end-points (cardiac-related hospitalization), and the optimal threshold of the VE/VCO_2_ slope was ≥32.9. Although β-blockers have been shown to improve the outcomes of patients with CHF, the results of the study by Arena *et al* (34) indicated that β-blockers have no influence on the prognostic value/characteristics of the VE/VCO_2_ slope. In the present study, the influence of β-blockers should be taken into account.

In conclusion, the present results indicate that ventilatory efficiency decreases in Chinese patients with CHF and support the proposal that the VE/VCO_2_ slope is a valuable prognostic indicator in Chinese patients with CHF.

## Figures and Tables

**Table I:** Baseline characteristics for each group of participants.

Characteristic	CHF group	Control group	P-value
n	129	129	
Age, years	59.1±11.4	56.8±8.8	0.068
Gender, M/F	113/16	106/23	0.297
BMI, kg/m^2^	24.7±3.7	24.5±3.0	0.807
LVMI, g/m^2^	138.8±46.5	100.7±15.8	0.001
LVEF, %	38±9	69±4	<0.001
NYHA grade, I/II/III	5/68/56	129/0/0	<0.001
β-blockers, n (%)	115 (89.0)	0	<0.001
ACEI or ARB, n (%)	117 (91.0)	0	<0.001
Diuretics, n (%)	66 (51.0)	0	<0.001
Digoxin, n (%)	55 (43.0)	0	<0.001
Peak VO_2_, ml/kg/min	14.0±3.9	22.2±4.2	<0.001
VE/VCO_2_ slope	38.9±8.7	28.6±3.9	<0.001

Data are expressed as the mean ± standard deviation, unless otherwise stated. CHF, chronic heart failure; M/F, male/female; BMI, body mass index; LVMI, left ventricular mass index; LVEF, left ventricular ejection fraction; NYHA, New York Heart Function Assessment; ACEI, angiotensin-converting enzyme inhibitor; ARB, angiotensin receptor blocker; peak VO_2_, peak oxygen consumption; VE/VCO_2_, minute ventilation/carbon dioxide production.

**Table II:** Receiver operating characteristic curve analysis of the VE/VCO_2_ slope for cardiac-related events in patients with chronic heart failure.

End-point	AUC of the VE/VCO_2_ slope	Optimal threshold	Sensitivity	Specificity	P-value	AUC 95% CI
Cardiac-related mortalities	0.670	≥39.3	0.667	0.620	0.021	0.549–0.790
Cardiac-related hospitalizations	0.682	≥32.9	0.631	0.778	0.045	0.485–0.798

VE/VCO_2_, minute ventilation/carbon dioxide production; peak VO_2_, peak oxygen consumption; AUC, area under the curve; CI, confidence interval.

**Table III:** Univariate Cox regression analysis for the VE/VCO_2_ slope in patients with chronic heart failure.

Variable	Optimal threshold	HR	P-value	HR 95% CI
VE/VCO_2_ slope	≥39.3	1.38	0.017	1.14–2.28
VE/VCO_2_ slope	≥32.9	0.71	0.012	0.41–0.97

VE/VCO_2_, minute ventilation/carbon dioxide production; HR, hazard ratio.

## Abbreviations

CHF: chronic heart failure
CPET: cardiopulmonary exercise testing
peak VO_2_: peak oxygen consumption
VE/VCO_2_: minute ventilation/carbon dioxide production
BMI: body mass index
LVMI: left ventricular mass index
LVEF: left ventricular ejection fraction
NYHA: New York Heart Function Assessment
ACEI: angiotensin-converting enzyme inhibitor
